# Use of mannans as an elicitor of the defense response on *Vitis vinifera* against fungi causing Grapevine Trunk Diseases

**DOI:** 10.1371/journal.pone.0343013

**Published:** 2026-02-17

**Authors:** Alfonso Ortega, José Antonio García, Jerónimo del Moral, Francisco Espinosa, Inmaculada Garrido

**Affiliations:** 1 Grupo de Investigación Fisiología y Biología Celular y Molecular de Plantas (FBCMP), INURA, Universidad de Extremadura, Badajoz, Spain,; 2 Centro Tecnológico Nacional Agroalimentario “Extremadura” (CTAEX), Ctra. Villafranco-Balboa 1.2, Badajoz, Spain; 3 Grupo de Investigación Calidad y Microbiología de los Alimentos (AGAO17), INURA, Universidad de Extremadura, Badajoz, Spain; University of Duhok, IRAQ

## Abstract

Grapevine Trunk Diseases (GTDs) are caused by phytopathogenic fungi that compromise grapevine productivity and wine quality. Most GTDs preventive treatments are chemical-based and environmentally harmful. One goal of the European Green Deal is to develop sustainable agriculture which does not harm the environment and reduces pesticide use and an alternative to those treatments may be the use of elicitors such as oligosaccharides from fungi. Many studies confirm that oligosaccharides activate the defence response. The experiment was carried out in vineyards of Tempranillo and Airén cvs. Asymptomatic and symptomatic vines were treated with mannans. Leaves and grapes were taken and pigments and phenols content, polyphenol oxidase (PPO) and superoxide dismutase (SOD) activities and gene expression of several defence enzymes were determined. The mannan addition to symptomatic vines was more positive for the leaves than for the grapes, palliating the damage caused by the disease, especially in the cv. Tempranillo. On the one hand, in the leaves, mannans caused an increase in phenols and PPO activity and expression; on the other hand, in grapes, although phenols increased, the other parameters did not. Mannans increased the expression levels of chalcone synthase (*CHS1*, *CHS3*), phenylalanine ammonia lyase (*PAL*), *SOD*, and *PPO* in asymptomatic leaves of both cultivars. In symptomatic leaves, *CHS3* and *PAL* expression decreased in both cultivars, while *CHS1* and *PPO* increased only in Tempranillo. In grapes, the expression of the genes varied due to the development of the disease. The mannan treatment seemed to reduce the oxidative stress caused by GTDs, but, above all, mannans would act as a biostimulant activaing the defence system of asymptomatic vines that would help them respond more successfully to a possible pathogenic fungi infection, that although this response depended on the cultivar.

## 1. Introduction

Given the outlook of climate change, growing populations and the need to safely and sustainably respond to safely and sustainably to current and future food demands, it is crucial to develop new approaches that can better protect crops against biotic and abiotic stressors [1].

*Vitis vinifera L.* is a crop with a worldwide distribution and has a major impact on many countries’ economies. One of the main problems facing viticulture today derive from climate change or from grapevine trunk diseases (GTDs). These diseases are currently considered of the most destructive of this crop and compromise not only vine longevity and productivity but also wine quality [2], with the result being economic losses due to increased production costs [3]. GTDs are a group of vascular diseases produced by numerous pathogenic fungi, which affect grapevine wood and inhabit the xylem cells in the woody tissue [4,5]. When this type of fungus colonizes this tissue, it causes deterioration of the host plant due to the loss of xylem function and the consequent decrease in hydraulic conductivity [6]. Moreover, pathogenic fungi can produce compounds that activate secondary metabolism and enzymatic reactions, [7,8]. By 2018 already, 133 species had been identified belonging to 34 different genera [3]. Due to this great variety of pathogenic fungi involved in GTDs, the infection process and the external and internal symptoms can range fairly widely, depending on the pathology being analysed [3,4], although there is generally necrosis and decay in the wood tissue leading over time to foliar symptoms and the plant’s death [6].

In vineyards in Castilla-La Mancha, where this research was conducted, Tolosa-Almendros et al. [9] reported an increase in the incidence of these diseases caused by *P. chlamydospora*, *Phaeoacremonium spp.* (causing esca and Petri disease), *D. seriata* (responsible for Botryosphaeria dieback), and Cylindrocarpon-like anamorphs (causing black-foot disease). These fungal organisms were found to be the most abundant in this location, with their associated diseases being the most prevalent. Tolosa-Almendros et al. [9] also highlights the need to find efficient and sustainable measures to control these types of infections.

Alternatives are currently being sought to reduce the impact of some GTDs without the use of pesticides, as this is one of the objectives included in the European Green Deal. One such alternative could be the use of elicitors such as carbohydrates, compounds with no toxicity, which can be extracted from renewable sources and contribute to conserving the environment [10]. Many studies have confirmed that oligosaccharides play some important roles in activating plant innate immunity. The mannan oligosaccharides (MOS) are a type of saccharide derived from the cell wall of the yeast *Saccharomyces cerevisiae*. The use of MOS as elicitor could be a valuable strategy for controlling plant disease since they have been reported to induce local defences and systemic resistance to pathogens [11].

Yeast cell-wall extracts contain several compounds (mannoproteins, glucans, and chitin) that can act as elicitors triggering different defence modes in plants [12,13]. They are non-toxic, biodegradable, and ecological in the sense of being environmentally friendly. Treatment with them increases the activity of chitinase, a member of the group of enzymes related to pathogenesis, i.e., it is induced by pathogen infection. Plants’ main response to fungus attack is to secrete chitinase because of its strong antifungi potential, degrading the fungus’s cell wall [14]. Application of these extracts in the field can increase the plants’ growth and yield [15] as well as the phenol content, antioxidant activity, and concentration of photosynthetic pigments [16]. The use of cell wall extracts, with a large proportion of the compounds they contain being mannans, increases resistance to fungi and bacterial infections. In grapes, the anthocyanin and stilbene contents increase [13], while foliar application alters the anthocyanin content but not flavonoids content [12]. Mannan extracts have the capacity to eliminate reactive oxygen species (ROS) [17], and in the vine their application protects against another fungi disease – downy mildew– modifying the metabolism of phenylpropanoids and phytohormones, enzymes such as superoxide dismutase (SOD) and polyphenol oxidase (PPO), and the photosynthesis process [18]. With the application of these extracts, there is a physiological alteration involving reduced free and total amino acid contents, and resistance to the disease is induced in cv. Tempranillo [13,19].

Our main goal is to make an approach of how symptomatic or asymptomatic grapevine plants for GTDs behave after being treated with an extract of *S. cerevisiae*. This extract contains mannans, a component of the cell wall of *S. cerevisiae*, and would act as an elicitor that triggers a defense response in plants. In this way, the plant defense mechanisms would already be prepared for a possible attack by pathogenic fungi, being a more sustainable mechanism than the use of pesticides or other toxic agents. For it, photosynthetic pigments, phenolic compounds and activity of enzymes (SOD and PPO) involved in defense processes have been measured in two cultivars of grapevine plants: cv. Airén and cv. Tempranillo. Furthermore, the expression patterns of enzymes chalcone synthase (*CHS1, CHS2*) and phenylalanine ammonia lyase (*PAL*) involved in the biosynthesis of phenolic compounds, in the oxidation of phenols to quinones (PPO) and in the detoxifying activities of superoxide anion (SOD) were quantified.

## 2. Materials and methods

### 2.1. Plant material and growing conditions

The experiment was carried out in vineyards located in Carrilejo-Manzanares (X:39.022277, Y:3.388166) and Daimiel (X:39.133228, Y:3.467667) in the province of Ciudad Real for cv. Tempranillo and cv. Airén (*Vitis vinifera* L.), respectively. The cv. Tempranillo vineyard (5 ha) had been planted in 2000 with a 2.0 m × 2.9 m frame (1724 vines ha^-1^), and that of cv. Airén (6 ha) in 1996 with a 2.6 m × 2.6 m frame (1479 vines ha^-1^). The climate conditions are shown in Table 1 (obtained from the “Agroclimatic Information System for Irrigation”. Ministry of Agriculture, Fisheries and Food. Government of Spain).

**Table 1:** Climatic conditions during the 2020 (Manzanares/Daimiel weather station).

Month	T_min_ (°C)	T_max_ (°C)	T_mean_ (°C)	RH %	Radiation (MJ m^-2^ day^-1^)	Rainfall (mm)
January	−6.31/-5.64	15.75/15.57	4.78/4.81	94.69/86.66	7.29/7.32	29.85/17.05
February	−2.41/-4.04	21.52/21.35	8.66/8.18	86.60/82.95	11.47/11.51	5.96/4.13
March	−2.01/-2.78	28.09/27.07	10.00/9.51	74.63/75.10	13.59/14.94	39.40/34.40
April	1.68/1.42	23.33/23.89	12.99/12.51	81.86/78.86	16.30/17.26	54.10/38.40
May	6.32/5.69	35.53/34.46	19.05/18.78	62.03/58.45	22.72/24.41	43.51/36.40
June	6.32/7.29	39.66/38.37	22.44/22.22	42.21/39.93	26.88/28.73	0.98/2.00
July	14.10/13.47	41.40/41.88	27.17/27.94	43.60/34.90	27.57/27.90	29.40/1.80
August	6.85/7.15	39.85/40.15	24.83/25.27	41.93/36.85	25.95/25.32	8.32/10.60
September	4.23/5.35	34.37/33.96	19.75/20.16	57.87/51.25	19.91/18.61	26.85/18.20
October	−1.94/-0.04	29.88/28.72	12.99/13.04	68.84/63.80	15.21/13.65	26.88/16.40
November	−4.70/-4.04	24.97/24.00	9.93/9.48	88.38/87.15	8.78/7.20	47.90/70.60
December	−4.09/-6.11	14.82/14.05	6.16/5.46	91.09/90.40	7.29/6.00	30.38/25.00

Monthly mean temperatures (T_min_, T_max_ and T_mean_), relative air humidity (RH), radiation and rainfall. The shading shows the months of treatment with mannans until harvest.

The degree of expresion of disease of the analysed grapevines was determined using the McKinney index [20]. This method was developed by the scientist McKinney in 1923 and is the most widely used in plant pathology to quantify the severity of diseases in plants, including grapevines affected by grapevine trunk diseases (GTDs) [21]. The McKinney index is based on estimating the proportion of tissue with symptoms of the disease in a plant or group of plants, enabling researchers and growers to obtain a quantitative measure of disease severity and assess the effectiveness of treatments or management practices. Its scale ranges from 0 to 4, with 0 for asymptomatic plants; 1 grapevines with mild symptoms; 2 plants with moderate symptoms; 3 grapevines with severe symptoms; 4 plants dead due to the expresion of disease. For this research, asymptomatic plants (A, value of 0 in the McKinney index and showing no GTDs symptoms from the plantation, at least the last 5 years) and symptomatic plants (S) with moderate and severe symptoms (2–3 in the McKinney index, with visible GTDs symptoms like necrotic leaf lesions and berries wilt, at least the last 5 years) were used.

Asymptomatic vines (A) and symptomatic vines (S) were subjected to treatments with a mannans extract obtained from the cell wall of *Saccharomyces cerevisiae* SP1026, which was provided by “Alltech Crop Science Iberia SL, Molina de Segura, Murcia (Spain)”*.* The experimental design was randomized block with 4 replications (132 trunks per treatment). The mannan doses were performed from an experimental formulation (60 mg L^-1^), which has a cost of approximately 16 euros L^-1^. The treatments were 0, 1, 2, and 3 mL L^-1^ for asymptomatic (AT0, AT1, AT2, AT3) and symptomatic vines (ST0, ST1, ST2, ST3). A series of 8 foliar applications were applied, spraying 600 L of the mannan broth per ha. The initial application in Tempranillo was at the beginning of flowering (BBCH = 60–62 [22]) and in Airen the first application coincided with the setting of fruit (BBCH = 71), on May 7 and 21, respectively, and the last one around harvest time. Applications were made every 15 days, thus, in cv Tempranillo the dates were: 05/07; 05/21; 06/04; 06/18; 07/07; 07/22; 08/06 and 08/18 and cv Airén: 05/21; 06/04; 06/18; 07/07; 07/22; 08/06; 08/18 and 09/02. Leaf and grape samples were taken at harvest time (September 2 and 24 for Tempranillo and Airen respectively). Twenty leaves and 100 g of grapes per vine were chosen randomly from all sides and different parts of vine and clusters, respectively. The samples were taken from four vines from each of the four blocks (16 vines per treatment), at least 4 independent experiments were carried out with each of them.

The field work and the application of the different agronomic treatments were carried out thanks to a research agreement between the authors (University of Extremadura) and the owners of the vineyards and applied solutions (“Bodegas Yuntero” S.C.).

### 2.2. Photosynthetic pigment content

Leaf discs were taken from fresh leaves and incubated in methanol (0.0125 g mL^-1^) for 24 h in darkness at room temperature. The chlorophyll *a*, chlorophyll *b*, and carotenoid contents were determined spectrophotometrically in the methanolic extract by measuring A_666_, A_653_, and A_470_. The total chlorophyll and carotenoid contents were calculated in accordance with Wellburn [23]. The results are expressed as µg pigment g^-1^ FW (fresh weight).

### 2.3. Phenol, flavonoid and PPG content

Total phenols, flavonoids, and phenylpropanoid glycosides (PPG) were extracted from 1 g fresh leaves or grapes by homogenizing in methanol, chloroform, and 1% NaCl (5:5:2.5 mL), filtering, and centrifuging at 3200 *g* for 10 min. Total phenols were determined spectrophotometrically at A_765_ with the Folin-Ciocalteu reagent [24], expressing the result as μg caffeic acid g^-1^ FW from a standard curve of caffeic acid. Total flavonoid content was measured at A_415_ [25], and were calculated using the standard rutin curve and expressing the result as μg of rutin g^-1^ FW. Phenylpropanoid glycosides were determined at A_525_ [26], and were calculated on the basis on the standard verbascoside curve and expressing the result as μg of verbascoside g^-1^ FW.

### 2.4. Grape anthocyanin content

The anthocyanin content was measured in cv. Tempranillo grapes by the differential pH method. This is based on the structural transformation that anthocyanins undergo with changing pH (coloured at pH 1.0, colourless at pH 4.5) [27]. For this, 2.5 g of grapes were homogenized in 2.5 mL of an ethanol: 0.1 M HCl solution (85:15% v:v), followed by centrifuging at 4000 *g* for 10 min, diluting the supernatant in two different buffers (0.025 M KCl, pH 1.0, and 0.4 M sodium acetate, pH 4.5), and incubating for 30 min at room temperature. The absorbance at 520–700 nm was measured. The anthocyanin content was expressed as mg of malvidin 3-glucoside (a major grape pigment) g^-1^ FW, in accordance with the equation given by MohdMaidin et al. [28]. The anthocyanin content in cv. Airén was not quantified because, as mentioned in Polat et al. [29], the level of anthocyanins in this type of cv. was undetectable.

### 2.5. PPO and SOD activities

To measure the PPO activity, leaves (0.5 g mL^-1^) or grapes (1.5 g mL^-1^) were homogenized in phosphate buffer (100 mM, pH 6.5) with 1% polyvinylpolypyrrolidone (PVPP), centrifuged at 19 000 *g* for 30 min at 4 °C, and the supernatant was used for the enzyme determination. The protein content was determined by the Bradford method [30]. PPO activity was determined by measuring A_420_ at 25 °C in a medium containing the enzyme extract, 100 mM phosphate buffer, and 0.1 M catechol [31].

For SOD activity, fresh leaves (0.5 g mL^-1^) or grapes (1.5 g mL^-1^) were homogenized at 4 °C in 50 mM pH 6.0 phosphate buffer, 1 mM ethylenediaminetetraacetic acid (EDTA), 0.5 mM phenylmethylsulfonyl fluoride (PMSF), 1 mM β-mercaptoethanol, 1 g L^-1^ PVPP. The homogenate was filtered and centrifuged at 39 000 *g* for 30 min at 4 °C, and the supernatant was used for the enzyme determination. The protein content was determined by the Bradford method [30]. Superoxide dismutase (SOD) activity was determined at A_560_ in a medium containing 50 mM phosphate buffer pH 7.8, 0.1 mM EDTA, 1.3 μM riboflavin, 13 mM methionine, and 63 μM 4-nitro blue tetrazolium (NBT) [32].

### 2.6. RNA extraction and cDNA synthesis

The plant material (fresh leaves or grapes) was frozen in liquid nitrogen and stored at −80°C until RNA extraction. RNA was extracted and purified using the Spectrum Plant Total RNA kit (Sigma-Aldrich®) and RNase-Free DNase (Qiagen®, No 79254). To determine the concentration and purity of each RNA sample, an Eppendorf D30 biospectrometer (Eppendorf, Germany) was employed. Both parameters were assessed by measuring the wavelengths (λ) at 260/280 nm, as the maximum absorbance of nucleic acids is at 260 nm and the maximum absorbance of proteins is at 280 nm. Qualitatively, only samples with a 260/280 nm range between 1.8 and 2.1 were considered suitable.

The integrity of the extracted and purified nucleic acids was evaluated via electrophoresis on a 1% agarose gel in 1X Tris-acetate-EDTA (TAE) buffer. The electrophoresis results showed three bands for each sample: two well-defined bands corresponding to 28S RNA and 18S RNA, and a lower, diffuse band corresponding to small RNA, with no indication of degradation. The gel was visualised using a Geneflash transilluminator (Syngene®).

### 2.7. qRT-PCR

The real-time amplification was monitored with SYBR Green (Thermo Fisher Scientific) on a QuantStudio 1 amplification and detection instrument (Applied Biosystems, Thermo Fisher Scientific R). *VATP16* (V-type proton ATPase) and *ACTIN2* were used as housekeeping genes [33,34]. Previous studies have qualified both as being genes with a stable expression pattern under biotic stress conditions [35]. The primers used for grapevine cDNA amplification are given in Table 2.

**Table 2:** Oligonucleotides and information used for real-time quantitative RT-PCR analysis of *Actin2, SOD, PAL, PPO, CHS1, CHS3* and *VATP16* of grapevine cDNAs.

Gene	F/R	Sequence 5´-3´	Information	Size (bp)
***SOD***	F	CTGCGGGTTGGTGTTCTAAT	superoxide dismutase, chloroplastic/cytosolic VIT_02s0025g04830	156
	R	TTCCCATATGGTGGTTCCAT		
***PAL***	F	ACAACAATGGACTGCCATCA	Phenylalanine ammonia lyase VIT_16s0039g01300	192
	R	GGAGGAGATTAAGCCCAAGG		
***PPO***	F	GGCTTTTCTTCCCTTTCCAC	*V. vinifera* polyphenol oxidase, chloroplastic-like (LOC100261681), misc_RNA	205
	R	ATTACAGTCGGAGGCAGGTG		
***ACTIN2***	F	ACTGCTGAACGGGAAATTGT	*V. vinifera* actin 2 (act2) mRNA Actin2-S1 AF369525	189
	R	AGTCCTCTTCCAGCCATCT		
***CHS1***	F	AGCCAGTGAAGCAGGTAGCC	chalcone synthase (AB015872)	155
	R	GTGATCCGGAAGTAGTAAT		
***CHS3***	F	GTTTCGGACCAGGGCTCACT	chalcone synthase 3 (AB066274)	93
	R	GGCAAGTAAAGTGGAAACAG		
***VATP16***	F	CTTCTCCTGTATGGGAGCTG	V-type proton ATPase 16 kDa proteolipid subunit	112
	R	CCATAACAACTGGTACAATCGAC		

### 2.8. Statistical analysis

The means ± SD of each measurement are presented in each figure or table (from at least 4 independent experiments). For each measurement, a Shapiro–Wilk normality test was performed (since n < 50) followed by a one-way ANOVA parametric test. The values for which there are significant differences, i.e., where p ≤ 0.05, are marked with different letters. All statistical analyses were performed with Microsoft Excel 365 (Microsoft Inc., Alburquerque, NM, USA) and R version 2.9.2 (2009-08-24; Copyright (C) 2009 The R Foundation for Statistical Computing; ISBN 3-900051-07-0).

## 3. Results

### 3.1. Photosynthetic pigment content

The photosynthetic pigment contents of leaves of both cultivars were determined (Table 3). In asymptomatic cv. Tempranillo leaves, there were decreases in both chlorophyll *a* and chlorophyll *b* content in response to the application of mannans. These decreases increased with dose. In symptomatic leaves, the mannan application led to a decrease in the content of both chlorophylls relative to the asymptomatic leaf control values (AT0), but no such significant effect relative to the symptomatic leaf control values (ST0). In asymptomatic cv. Airén leaves, the application of the different mannan treatments did not alter the chlorophyll content but, in symptomatic leaves, there were significant decreases in the chlorophyll a with respect to the control values (ST0). In particular, there were slight dose-dependent decreases in chlorophyll *a* while the chlorophyll *b* content remained unaltered. For the same given treatment, symptomatic leaves showed lower chlorophyll *a* and *b* contents.

**Table 3:** Effects of treatment (T) of mannans (0 mg L^-1^: T0, 1 mg L^-1^: T1, 2 mg L^-1^: T2, and 3 mg L^-1^: T3) on the chlorophyll (chl) a and b, and total chlorophyll content, chlorophyll a/b ratio, total carotenoids (car) and carotenoid/chlorophyll ratio in leaves of cv. Tempranillo and Airen grapevines, asymptomatic (A) and symptomatic (S). Chlorophylls and carotenoids are expressed in µg g^-1^ FW. The data are means ±SD, and the same letters of each grapevine cultivar or AT and ST, indicate do not differ significantly.

cv	T	Chl a		Chl b		Chl a+b		Chl a/b	Car		Car/Chl	
Tempranillo	AT0	1723.4 ± 52.9^c^		1138.6 ± 87.1^c^		2861.9 ± 128.7^c^		1.52 ± 0.08^a^	100.0 ± 13.4^ab^		0.030 ± 0.008^a^	
	AT1	1560.5 ± 67.0^b^		909.1 ± 98.7^b^		2469.6 ± 165.5^b^		1.73 ± 0.11^b^	107.2 ± 16.0^ab^		0.044 ± 0.009^ab^	
	AT2	1448.4 ± 19.3^b^		784.4 ± 31.2^ab^		2232.8 ± 49.7^b^		1.85 ± 0.04^b^	123.0 ± 2.8^b^		0.055 ± 0.001^b^	
	AT3	1219.8 ± 74.2^a^		700.6 ± 73.3^b^		1920.4 ± 144.3^a^		1.75 ± 0.09^b^	95.2 ± 7.4^a^		0.050 ± 0.007^b^	
	ST0	996.9 ± 68.6^a^		439.7 ± 20.6^a^		1520.2 ± 104.8^a^		2.00 ± 0.10^bc^	116.2 ± 4.9ab		0.078 ± 0.006b	
	ST1	1134.7 ± 120.5^a^		589.2 ± 68.5^a^		1724.0 ± 187.2^a^		1.93 ± 0.06^b^	125.3 ± 12.0ab		0.074 ± 0.007b	
	ST2	973.2 ± 211.4^a^		485.1 ± 118.9^a^		1456.5 ± 331.5^a^		2.02 ± 0.07^c^	130.0 ± 12.5b		0.090 ± 0.019b	
	ST3	1020.4 ± 168.1^a^		627.6 ± 120.6^a^		1647.9 ± 273.8^a^		1.67 ± 0.19^a^	107.2 ± 11.2^a^		0.053 ± 0.007^a^	
Airén	AT0	1330.4 ± 70.1^a^		668.8 ± 26.3^a^		1999.2 ± 95.8^a^		1.99 ± 0.03^b^	117.6 ± 17.6^a^		0.060 ± 0.012^a^	
	AT1	1275.0 ± 244.3^a^		663.6 ± 163.2^a^		1938.6 ± 405.9^a^		1.94 ± 0.11^b^	118.6 ± 8.8^a^		0.064 ± 0.014^a^	
	AT2	1066.9 ± 92.4^a^		536.3 ± 49.6^a^		1603.2 ± 127.1^a^		2.00 ± 0.17^b^	114.4 ± 19.5^a^		0.071 ± 0.010^a^	
	AT3	1248.4 ± 208.0^a^		747.8 ± 129.0^a^		1996.2 ± 331.8^a^		1.67 ± 0.10^a^	84.5 ± 21.7^a^		0.044 ± 0.011^a^	
	ST0	868.8 ± 0.8^c^		405.6 ± 12.4^a^		1274.4 ± 12.0^b^		2.14 ± 0.06^a^	111.8 ± 11.7^b^		0.089 ± 0.011^a^	
	ST1	680.8 ± 85.8^b^		303.0 ± 38.3^a^		983.8 ± 123.5^a^		2.18 ± 0.14^a^	115.8 ± 20.6^b^		0.112 ± 0.023^a^	
	ST2	647.8 ± 115.1^b^		314.6 ± 70.7^a^		962.4 ± 183.8^a^		2.08 ± 0.13^a^	83.2 ± 12.6^a^		0.088 ± 0.016^a^	
	ST3	509.2 ± 111.5^a^		293.4 ± 112.8^a^		802.6 ± 216.1^a^		2.01 ± 0.25^a^	75.5 ± 11.6^a^		0.109 ± 0.042^a^	

The chlorophyll *a*/*b* ratio was calculated since its increase may indicate stress in plants (Table 3). For cv. Tempranillo, in asymptomatic leaves the mannan application led to an increase in this ratio, and it was also significantly higher in symptomatic leaves relative to healthy leaves (AT0). Mannan application induced a decrease in the *a*/*b* ratio in symptomatic leaves only for ST3. For cv. Airén, in asymptomatic leaves neither AT1 nor AT2 affected this ratio, with only AT3 leading to a significant decrease. In symptomatic leaves, this ratio was higher than in the asymptomatic leaves (ST0) and remained unaffected by the mannan treatments.

With respect to the carotenoid content, in Tempranillo (Table 3), slight oscillations with little significance were observed in both asymptomatic and symptomatic leaves due to the effect of mannan application. Neither were there changes in the carotenoid content in AT0 and ST0. One was observed that the ratio between carotenoids and total chlorophylls increased significantly in healthy leaves when mannans were applied. In symptomatic leaves, the values were higher than in asymptomatic leaves, and at ST3 the mannan application led to their significant reduction. In cv. Airén, the carotenoid content of asymptomatic leaves was unaffected by mannan treatments. For symptomatic leaves, the carotenoid content was similar in ST0 to that in AT0, and the mannan treatments ST2 and ST3 significantly reduced their carotenoid contents. With respect to the ratio of carotenoids to total chlorophyll, mannan application led to no significant differences in either asymptomatic or symptomatic leaves, but the disease increased this ratio relative to the asymptomatic leaf case. This response of an increased carotenoid-to-chlorophyll ratio was observed in cv. Tempranillo in response both to mannans and to the disease, but in cv. Airén only in response to the disease.

### 3.2. Phenol, flavonoid and PPG content

Total phenol, total flavonoid, and total phenylpropanoid glycoside (PPG) contents were determined in leaves and grapes of asymptomatic and symptomatic vines of both cultivars, as also were the effects on these contents of the different mannan treatments used. The three groups of effects were similar.

**Fig 1:**
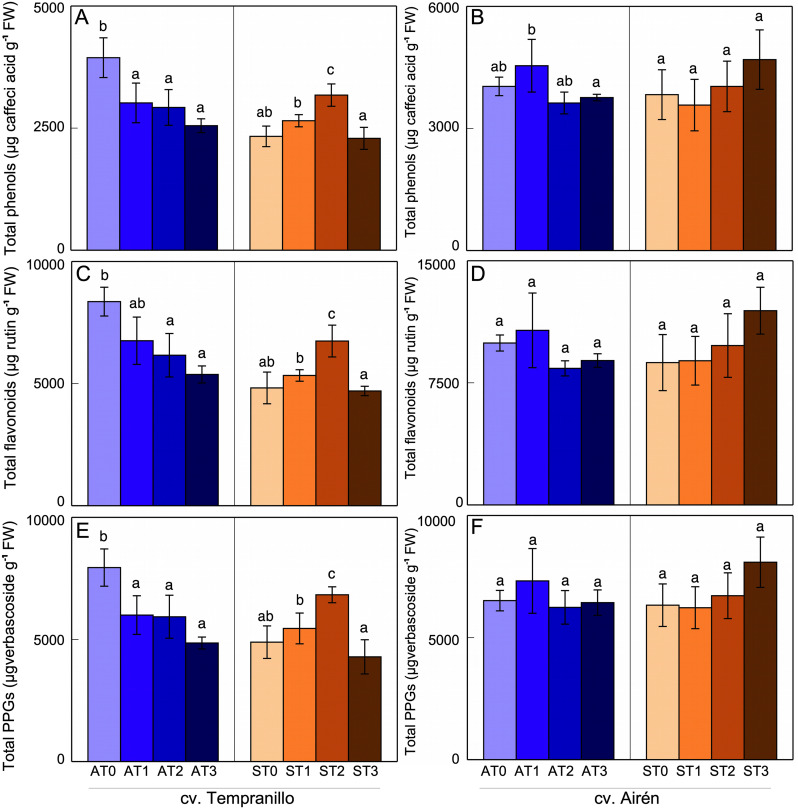
Total phenol (A,B) flavonoid (C,D) and PPGs (E,F) in leaves of the two grapevine cultivars asymptomatic (A) and symptomatic (S), treated with different doses of mannans (0 mg L^-1^: T0, 1 mg L^-1^: T1, 2 mg L^-1^: T2, and 3 mg L^-1^: T3). The data are means ±SD and bars of each grapevine cultivar marked with the same letter do not differ significantly according to a parametric test of one-way ANOVA.

For cv. Tempranillo (Fig 1A, C, E), in asymptomatic leaves the mannans reduced the total contents of all three groups -Phenol, flavonoid and PPG-. In symptomatic leaves, however, relative to ST0 there were significant increases in these compounds in ST2 but hardly any differences in ST1 and ST3. As evidenced by the comparison of ST0 with AT0, the disease itself did lead to a decrease in the content of these compounds. For cv. Airén (Fig 1B, D, F), the different treatments had little effect on these compounds’ contents. Only the total phenol content in asymptomatic leaves for the dose rate of AT1 showed a slight, but no significant, increase from AT0. There were no differences in this response between asymptomatic and symptomatic leaves. The total content of these phenols was unaffected by the disease.

**Fig 2:**
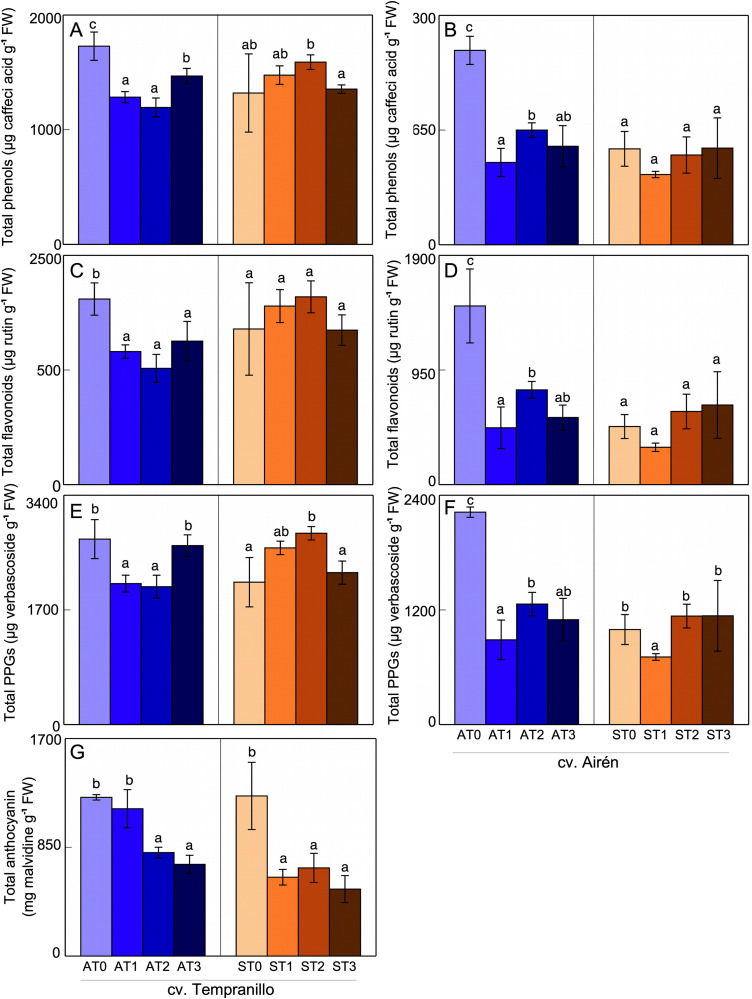
Total phenol (A,B) flavonoid (C,D), PPGs (E,F) and anthocyanin content (G) in grapes of the two grapevine cultivars asymptomatic (A) and symptomatic (S), treated with different doses of mannans (0 mg L^-1^: T0, 1 mg L^-1^: T1, 2 mg L^-1^: T2, and 3 mg L^-1^: T3). The data are means ±SD and bars of each grapevine cultivar marked with the same letter do not differ significantly according to a parametric test of one-way ANOVA.

In asymptomatic cv. Tempranillo grapes (Fig 2A, C, E), the mannans caused a decrease in the content of these compounds, the decrease being greater in AT1 and AT2 in the case of the PPGs. There were no differences between AT3 and AT0. In grapes from symptomatic vines, there were few differences between treatments, only worthy of note being significant increases in phenols and PPGs for ST2 relative to ST0. In the absence of mannans (AT0 and ST0), as a consequence of the disease, the total phenol, flavonoid and PPG contents decreased, but the anthocyanin content was similar. In asymptomatic cv. Airén grapes (Fig 2B, D, F), mannans strongly reduced the content of all these compounds. The response was somewhat different in grapes from symptomatic vines, with a decrease in ST1 relative to ST0 in the case of the PPGs, while in ST2 and ST3 the contents were similar to the control, ST0. The disease itself modified the total content of these compounds, as can be seen in the comparison of ST0 in AT0, with reductions in the content of all three groups – phenols, flavonoids, and PPGs.

In both asymptomatic and symptomatic cv. Tempranillo grapes, mannan application reduced the content of anthocyanin, the main flavonoid associated with red wine’s colour and astringency (Fig 2G). This content was high for both HT0 and DT0. In HT1, the decrease was non-significant (6%), but more pronounced and significant in HT2 and HT3. In symptomatic grapes, the decrease in anthocyanin content was strong, with the DT1, DT2, and DT3 values being similar to each other, and all lower than DT0. The disease itself did not alter the grape anthocyanin content since the HT0 and DT0 values were very similar.

For cv. Tempranillo, in leaves and grapes of asymptomatic vines, the addition of mannans negatively affected their phenol content. On the contrary, in symptomatic leaves, treatments ST1 and ST2 showed recovery of the content of these compounds to values similar to those of asymptomatic vines. Thus, in grapes from mannan-treated vines, the disease caused an increase in the total phenol content, and in grapes from both asymptomatic and symptomatic vines, the mannans caused a decrease in their anthocyanin content. For cv. Airén however, the mannan treatments had little effect in leaves, whether asymptomatic or symptomatic, and in grapes led to a decrease in the asymptomatic case, but hardly any effect in the symptomatic case.

### 3.3. Polyphenol oxidase (PPO) and Superoxide dismutase (SOD) activities

**Fig 3:**
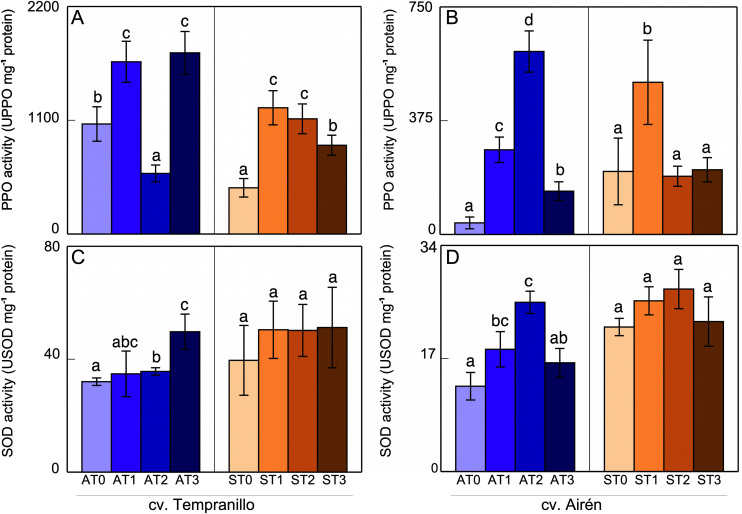
PPO (A,B) and SOD (C,D) activities in leaves of the two grapevine cultivars asymptomatic (A) and symptomatic (S), treated with different doses of mannans (0 mg L^-1^: T0, 1 mg L^-1^: T1, 2 mg L^-1^: T2, and 3 mg L^-1^: T3). The data are means ±SD and bars of each grapevine cultivar marked with the same letter do not differ significantly according to a parametric test of one-way ANOVA.

**Fig 4:**
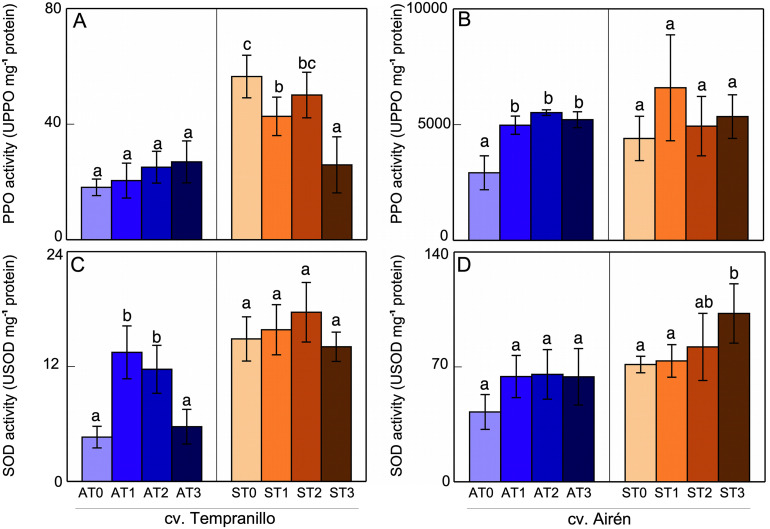
PPO (A,B) and SOD (C,D) activities in grapes of the two grapevine cultivars s asymptomatic (A) and symptomatic (S), treated with different doses of mannans (0 mg L^-1^: T0, 1 mg L^-1^: T1, 2 mg L^-1^: T2, and 3 mg L^-1^: T3). Bars of each grapevine cultivar marked with the same letter do not differ significantly according to a parametric test of one-way ANOVA.

Figs 3 and 4 show the PPO and SOD activities in leaves and grapes, respectively. With respect to the cv. Tempranillo leaves (Fig 3A), in the asymptomatic leaf case, the addition of mannans increased the PPO activity but not in a dose-dependent manner (AT1 and AT3 were similar, although the values were surprisingly low in AT2, this last result can be considered anomalous and difficult to explain). In the symptomatic leaf case, the addition of mannans also increased this activity, with that of ST0 being lower than that of AT0, evidence of a clear negative effect of the disease. With respect to the cv. Airén leaves, in the asymptomatic case (Fig 3B), PPO activity increased markedly in AT1 and AT2 (a dose-dependent increase), to then decrease in AT3, although with activity levels that were still higher than the control, AT0. In symptomatic leaves, only ST1 led to increased PPO activity compared with ST0, with ST2 and ST3 leaves presenting values similar to ST0. Thus, one can see that the effect is dependent on the dose in a non-trivial way, as there are instances in which a larger dose causes a decrease in the effect of mannans. The disease (ST0) induced increased PPO activity relative to the asymptomatic case (AT0), although with there being great variability in these increases.

The SOD activity in cv. Tempranillo leaves (Fig 3C) increased after the application of mannan to asymptomatic vines. In leaves from symptomatic vines, mannans did not seem to affect this activity. Likewise for cv. Airén, the asymptomatic leaves (Fig 3D) showed SOD activity in response to the increase in the amount of mannans, except AT3. In AT3, the activity level was decreased with respect to the lower mannan concentrations, with a value similar to AT0, probably, as indicated above, due to a less favorable effect with high doses. In symptomatic leaves, the mannans did not to affect the SOD activity. Comparison of the SOD activity values in AT0 and ST0 leaves shows that disease induced an increase in this activity through increasing the amount of H_2_O_2_ by dismutation and elimination of the O_2_^-^ produced in response to the stress caused by the disease.

For cv. Tempranillo grapes (Fig 4A), in the asymptomatic case, the mannan application had no in PPO activity. On the contrary, in the case of symptomatic grapes, it led to decreased PPO activity, especially ST3. The disease itself induced increased PPO activity (ST0 vs AT0). For cv. Airén grapes (Fig 4B, the mannans induced increased PPO activity in asymptomatic grapes. Their application caused an apparent increase PPO activity in symptomatic grapes (ST1 vs ST0) but, because of the great variability in the response, this increase was not significant. For ST2 and ST3, no significant differences were observed relative to ST0. In the absence of mannans, symptomatic grapes (ST0) presented greater PPO activity than asymptomatic grapes (AT0).

In asymptomatic cv. Tempranillo grapes (Fig 4C), an increase in SOD activity was observed following the addition of the first two doses of mannans (191% and 153% for AT1 and AT2, respectively). For AT3, there was no significant difference relative to AT0 (once again, lower doses seem to favor the effect of mannans). In grapes from symptomatic vines, no significant differences were observed between ST1, ST2, and ST3 and the ST0 control. In cv. Airén grapes (Fig 4D), the SOD activity increased slightly, but not significantly, for those from asymptomatic vines in response to the mannan application. For those from symptomatic vines, while the first doses of mannan affected this activity little, ST3 caused significant activation. Comparing the levels of SOD activity in asymptomatic and symptomatic grapes of both cultivars without application of mannans (AT0 vs ST0), one can say that disease caused a significant increase in SOD activity.

### 3.4. Gene expression

**Fig 5:**
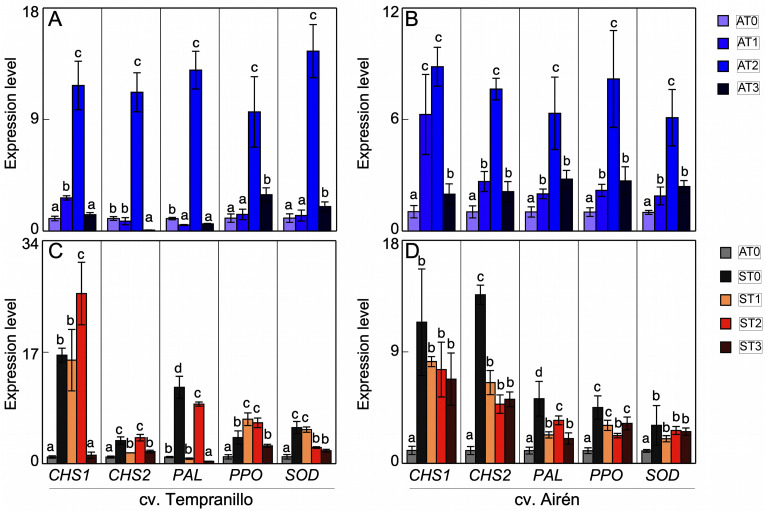
Expression level in leaves of CHS1, CHS2, PAL, PPO and SOD of two grapevine cultivars. Asymptomatic leaves of cvs. Tempranillo (A) and Airen (B) treated with different doses of mannans (0 mg L^-1^: AT0, 1 mg L^-1^: AT1, 2 mg L^-1^: AT2, and 3 mg L^-1^: AT3). Asymptomatic leaves of cvs. Tempranillo (C) and Airen (D) without treatment of manans (AT0) and symptomatic treated with the different doses of manans (0 mg L^-1^: ST0, 1 mg L^-1^: ST1, 2 mg L^-1^: ST2, and 3 mg L^-1^: ST3). Quantitative PCR in CHS1, CHS3, PAL, PPO and SOD (the same letter do not differ significantly according to a parametric test of one-way ANOVA).

The expression patterns of the *chalcone synthase* (*CHS1*, *CHS3*), *polyphenol ammonia-lyase* (*PAL*), *PPO*, and *SOD* genes were determined in leaves and grapes of both cultivars with the different treatments. Fig 5A–B shows the asymptomatic leaf results. In Tempranillo (Fig 5A), there was a very significant increase in the expression patterns of all the enzymes analysed with the AT2 treatment when compared with the untreated case. There was also a significant increase in the *CHS1* pattern in AT1 relative to the control plant, while in AT3 there was no difference with respect to AT0. *CHS3* in AT1 was expressed less than the control (AT0), and in AT3 there was practically no expression. The *PAL* expression also decreased in AT1 and AT3. However, the *PPO* and *SOD* expressions were activated in all treatments, although the difference between AT1 and AT0 was not significant. In Airén (Fig 5B), there were significant increases in the expression patterns of all the enzymes analysed with all treatments, especially in AT2 compared with AT0.

This increase in the PPO and SOD activities was also observed in the cv. Airén, as has been described above (Fig 3B and 3D). Again, the increase was greatest in AT2. There was not such an obvious relationship between expression and activity in cv. Tempranillo (Fig 3A and 3C), although the mannan treatments generally activated these activities in most cases, even if just slightly.

Analysing the changes in the expression level of *CHS1* and *CHS3*, *PAL*, *PPO*, and *SOD* of asymptomatic vines as against symptomatic vines without and with mannan treatments, it was clear that, both in Tempranillo (Fig 5C) and in Airén (Fig 5D), the symptomatic vines presented a significant increase in the expression pattern of all the enzymes studied when compared to the leaves of asymptomatic vines. In Tempranillo leaves, there was no correspondence between the expression and the measured activities. The disease caused a decrease in PPO activity (Fig 3A), while SOD activity was not affected (Fig 3C). However, in Airén leaves from symptomatic vines a correspondence was observed between the increases in activity and expression, both for PPO activity (Fig 3B), although it did not show significance, and for SOD activity (Fig 3D).

In Tempranillo, treatment with the highest dose of mannans (ST3) always mitigated the increase in expression caused by the infection in all the enzymes measured. With the treatments ST1 and ST2, the results differed according to the enzyme. In Airén, the foliar treatment with mannans seemed capable of alleviating the stress caused by GTDs. In particular, the expression level of the enzymes evaluated in treated symptomatic-GTDs vines was still significantly higher than that of asymptomatic vines, but lower than that found in untreated symptomatic vines. The mitigating effect of mannans was not seen in the enzymatic activities measured in symptomatic leaves of cv. Tempranillo: PPO increased due to the effect of mannans (Fig 3A) and SOD was unaltered (Fig 3C). Similar results were obtained in Airén (Fig 3B and 3D). The lack of correspondence between activity and expression described in some cases could be due to post-transcriptional regulations or external factors, which would directly affect enzymatic activity.

**Fig 6:**
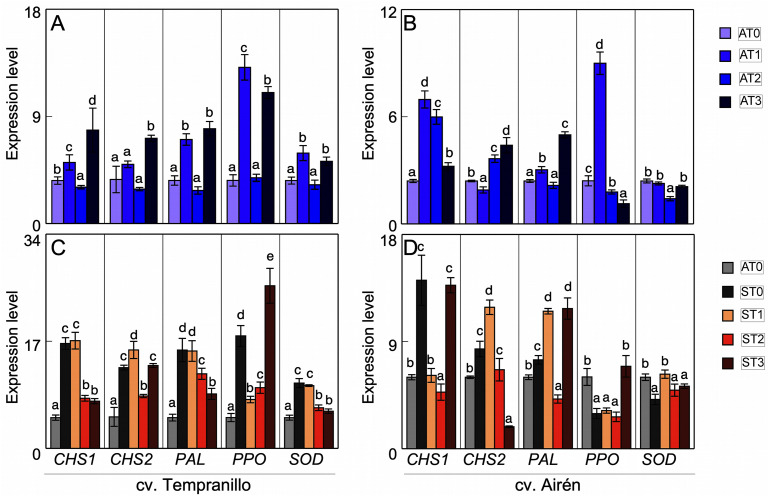
Expression level in grapes of CHS1, CHS2, PAL, PPO and SOD of two grapevine cultivars. Asymptomatic grapes of cvs. Tempranillo (A) and Airen (B) treated with different doses of mannans (0 mg L^-1^: AT0, 1 mg L^-1^: AT1, 2 mg L^-1^: AT2, and 3 mg L^-1^: AT3). Asymptomatic grapes of cvs. Tempranillo (C) and Airen (D) without treatment of manans (AT0) and symptomatic treated with the different doses of manans (0 mg L^-1^: ST0, 1 mg L^-1^: ST1, 2 mg L^-1^: ST2, and 3 mg L^-1^: ST3). Quantitative PCR in CHS1, CHS3, PAL, PPO and SOD (the same letter do not differ significantly according to a parametric test of one-way ANOVA).

The expression patterns of these enzymes in grapes from asymptomatic vines of both cultivars with and without mannan treatment differed from those obtained in leaves. In Tempranillo (Fig 6A), all the enzymes measured in grapes with the AT1 and AT3 treatments showed increased expression with respect to AT0 with one exception being that there was no significant difference between AT1 and AT0 in the expression of *CHS3*. With AT2 however, there was a decrease in *CHS1* expression relative to the control, but no such difference for *CHS3*, *PAL*, *PPO*, or *SOD*. In Airén (Fig 6B), each enzyme had a different response pattern to the different mannan doses. Thus, *CHS1* had increased expression in all treatments, but to a greater extent as the amount of mannans provided was lower. *CHS* expression decreased slightly in AT1, but increased in AT2 and AT3. *PAL* expression increased slightly in AT1, decreased in AT2 (to close to the AT0 value), and increased considerably in AT3. *PPO* expression increased in AT1 but decreased in AT2 and AT3 relative to AT0. *SOD* expression was practically unaffected in AT1 and AT3, but decreased in AT2.

Comparing the *PPO* and *SOD* expression results with those of these enzymes’ activities described above, one observed that in asymptomatic Tempranillo grapes the PPO activity (Fig 4A) was not significantly affected by mannans, but the expression was. The SOD activity (Fig 4C) showed a closer relationship with what was obtained in gene expression, with in general there being activation in both cases. In Airén, the maximum *PPO* expression was in AT1, decreasing in AT2 and AT3. The activation of PPO activity by mannans (Fig 4B) was similar in all treatments. Neither the activity nor the expression of *SOD* (Fig 4D) was affected by mannan treatment.

Fig 6C, D shows the expression of the enzymes *CHS1* and *3*, *PAL*, *PPO*, and *SOD* in grapes from GTDs-affected vines with and without mannan application versus grapes from asymptomatic vines. For Tempranillo (Fig 6C), the expression was greater in symptomatic vines, with or without mannan application, than in control vines. In this, the ST1 expression values were similar to those of untreated diseased vines except for *PPO* where ST1 expression fell significantly. ST2 decreased the expression of all the enzymes studied, although without reaching the levels of asymptomatic grapes. In ST3, the results depended on the enzyme: the expression levels of *CHS1*, *PAL*, and *SOD* fell compared with the symptomatic grapes, the *CHS3* expression levels were similar to those of diseased grapes, ST0, and there was a considerable increase in the case of *PPO*. In Airén (Fig 6D), the expression levels in symptomatic grapes did not increase in all cases relative to those of asymptomatic grapes. Instead, the said levels decreased for *PPO* and *SOD*. The results obtained with mannan treatments were very variable. Thus, ST1 increased *CHS3*, *PAL*, and *SOD* expression relative to untreated symptomatic grapes (ST0), decreased that of *CHS1*, and left that of *PPO* unchanged. Regarding ST2 relative to ST0, there was a decrease in the expression of *CHS1*and *PAL*, and there were no significant changes in the other enzymes. Finally, relative to ST1 and ST2, ST3 induced increased expression except for *CHS3* whose expression declined considerably, and, relative to ST0, it decreased the expression of *CHS3*, increased that of *PAL* and *PPO*, and left that of *CHS1* and *SOD* unchanged.

The expression of *PPO* in Tempranillo grapes was comparable with the results obtained for the activity of this enzyme (Fig 4A). An increase in activity, as in expression, was observed in symptomatic ST0 grapes relative to asymptomatic AT0 ones. Treatment with mannans lessened both this increase and the expression in ST1 and ST2. Only ST3 caused a decrease in activity, while the gene expression was very high. In Airén grapes, there was little parallelism between *PPO* expression and activity (Fig 4B). The disease caused an increase in activity that did not correspond to an increase in expression relative to asymptomatic grapes. The ST1 and ST2 mannan treatments affected neither the expression nor the activity with respect to ST0, while ST3 induced an increase in *PPO* expression but not in activity.

Regarding the *SOD* expression and activity of Tempranillo grapes (Fig 4C), the symptomatic vines showed an increase in both compared with asymptomatic grapes. Mannan treatment did not change the activity compared with untreated symptomatic grapes, but it did cause a decrease in expression in ST2 and ST3 (ST1 was unaffected). In Airén, while the symptomatic vines showed activation of SOD activity (Fig 4D), this was not due to increased expression of the enzyme. Mannan treatment increased both expression and activity relative to untreated symptomatic grapes.

## 4. Discussion

### 4.1. Photosynthetic pigment content

Mannan treatment negatively affects chlorophyll content in asymptomatic cv. Tempranillo vines; but does not affect symptomatic vines. In cv. Airén, mannans did not alter pigment content in asymptomatic vines, but caused a decrease in chlorophyll a content in symptomatic vines. Furthermore, the chlorophyll a/b ratio is not affected in cv. Airén and increases in cv. Tempranillo, which would indicate that both varieties behave differently, with Tempranillo being more sensitive to both the disease and mannans addition. The increase in the chlorophyll a/b ratio would indicate reduced thylakoid appression, which, according to Zhou et al. [36], might contribute to the dissipation of excess energy that would imply an adaptation to stress. Thus, in cv. Tempranillo both the disease and the mannans would cause stress in the photosynthetic apparatus. Only the highest mannan concentration showed a positive effect on the leaves of asymptomatic vines. In cv. Airén only the disease showed a negative effect, while mannans had practically no effect except for AT3 in which they had a protective effect.

In general, similar results regarding photosynthetic pigment content had been obtained by Petit et al. [37] who described a decrease in chlorophylls a and b in leaves of cv. Chardonnay affected by esca while carotenoids seemed to be unaltered and could have, according to those workers, a protective role for plant membranes against fungi in both symptomatic and asymptomatic leaves. Those authors reported no differences in the chlorophyll a/b ratio between asymptomatic and symptomatic leaves, apparently because the two chlorophylls were damaged in the same way. García et al. [8] described similar results for cv. Tempranillo, with a decrease in chlorophyll content in vines affected by esca, with increases in the chlorophyll a/b and no variation due to the effect of esca in the carotenoids in the vines harvested in August but an increased carotenoid/chlorophyll ratio. Also for cv. Tempranillo, Martin et al. [38] described decreases in symptomatic leaves in chlorophylls and in carotenoids. The carotenoid increase could be indicative of these compounds’ protective role because of their antioxidant activity. Santos et al. [39] also observed decreased chlorophyll a and b contents in infected vines, with an increased chlorophyll a/b ratio, the behaviour depending on the cultivar studied. Different results showed Dawood et al. [16] who observed increases in all photosynthetic pigments following the application of yeast extract to soybean plants. De Miccolis Angelini et al. [18] described the application of yeast extract to grapevines as having a strong enhancing effect on the expression of genes that encode enzymes related to photosynthesis processes.

### 4.2. Phenol, flavonoid and PPG content

The response to the addition of mannans or to infection was a decrease in the content of the different phenolic compounds, this does not, in general, coincide with the effects described by other workers for different elicitors. Thus, Sak et al. [40], applying methyl-jasmonate (MeJA) or *P. chlamydospora* in vine callus cultures, described an increase in the production of polyphenols, this effect being much greater with the fungi elicitor. García-Pastor et al. [41] also described increase ripening, harvest, total phenols, and particularly anthocyanins following the application of low concentrations of MeJA to cv. Magenta and Crimson grapes. An increase in phenols has also been observed in grape cell cultures elicited with two fungi (*Eutypa lata* and *Trichoderma atroviride*) [42]. Calzarano et al. [43] and Lima et al. [44] detected an increase in polyphenols in diseased vines. In general, the use of yeast in plants seemed to cause an increase in phenol content [18]. However, decreased phenol content, due to the effect of esca, had already been described by other workers [8,45]. This could be due to the involvement of phenols in defence reactions, being derived for the synthesis of compounds such as lignin [46]. Sgherri et al. [47] described a decrease in phenols in *Ramonda serbica* in response to water stress, possibly because these compounds would play an important role against the oxidation caused by the stress, which coincided with our results.

Overall one should take into account the time point were the phenolic compounds levels were measured, since their content can be greatly influenced by the phenological state [48].

With respect to grape anthocyanin content, Lorrain et al. [45] described a slight decrease in cv. Cabernet Sauvignon grapes from vines affected by esca. García et al. [8], however, indicated that esca does not affect the anthocyanin content in cv. Tempranillo grapes, which results that coincide with those of the present work in ST0. The application of mannans did affect the anthocyanin content causing it to decrease, possibly due to an effect on the route of synthesis of the flavonoids themselves responsible for the synthesis of tannins and anthocyanins, giving rise to a lower content of phenols.

### 4.3. Polyphenol oxidase (PPO) and Superoxide dismutase (SOD) activities

Previous studies have described increased PPO activity in grapevines affected by esca [8,49] and, as in this case, in grapes from diseased vines [8,50]. Rusjan et al. [51] described an increase in PPO activity in vine leaves of cv. Chardonnay as a consequence of Bois Noir infection. Shi et al. [52] in Cabernet Sauvignon described an increase in PPO activity due to *Colomerus viti*s infection, although this activation decreased as the infection develops. Similarly, our results showed that a decrease in PPO activity occured in leaves due to the effect of the disease, which could be due to the disease being in an advanced stage of development.

The addition of mannans to asymptomatic vines caused an increase in PPO activity, which would indicate the development of the defence reaction. Singh et al. [53] described a similar response after the application of chitosan to two grapevine cultivars, with an increase in the expression of PPO genes in both leaves and grapes. In practically all the cases that have been reported previously, the effect of mannans in asymptomatic vines was to increase SOD activity as an indicator of stress. This increase allows excess ROS to be controlled and eliminated [17]. Disease and other stressors induced SOD activation in grapevine leaves [54,55]. Zou et al. [56] described how the application of mannans from *Bacillus velenzensis* caused an increase in enzymatic activities related to the metabolism of the antioxidant glutathione (glutathione reductase and glutathione-S transferase) in soybean plants with salt stress.

The finding that in Airén leaves and Tempranillo grapes SOD activity increased at lower mannan concentrations and decreased at higher concentrations could be compared with the results of Shi et al. [52] for Cabernet Sauvignon in which the increased SOD activity in response to *Colomerus viti*s infection declined in more advanced stages of the infection.

### 4.4. Gene expression

Flavonoids belong to a huge group of phenolic plant constituents [57] and play an important role in tolerance to both abiotic and biotic stress, especially for its antioxidant capacity [58,59]. Enzymes involved in its metabolism (*CHS*, *PAL*) increase their expression by the addition of mannans or by infection. Recently, García et al. [8] was reported that these enzymes increased their expression pattern in leaves and fruits of grapevine plants with esca compared to asymptomatic grapevines. The results obtained were consistent with what was mentioned above, since symptomatic or asymptomatic vines treated with mannans showed a higher level of expression of these genes than healthy plants without any treatment. In addition, the attack of pathogenic fungi that trigger some GTD increases the expression of *CHS1* and *CHS3*, *PAL* and *SOD* since it involved generating an antioxidant response. PPO, although it participated in the degradation of phenolic compounds, also showed greater activity and expression level in asymptomatic vines treated with mannans or vines with symptoms of some GTDs. This fact is because the PPO activity would be involved in the resistance of the plant through the production of metabolites that were toxic for the pathogens, such as phytoalexins, phenols, and lignin. In addition, there were severe studies in vines and other plant species where it has been reported that PPO increased its activity and expression when attacked by pathogenic fungi such as *Fusarium solani* or fungi responsible for esca-diseased [50,60].

These expression results indicated that, with just a few exceptions, mannans caused increase expression of the enzymes measured in both leaves and grapes of asymptomatic vines. Furthermore, the expression of these enzymes was greater in symptomatic vines than in asymptomatic ones, and a consequence of the addition of mannans to diseased vines was in most cases to lessen the increase in the expression of these genes. These results agree with De Miccolis Angelini et al. [18] indicated that a yeast derivative (*S. cerevisane*) was effective against mildew in grapevines, and produced an increase in various genes including those, such as *PAL* and *SOD*, which encode proteins related to pathogenesis. Chitosan also had been found to activate *PAL*, *PPO*, and *SOD* genes in two grapevine cultivars in both their leaves and grapes [53,61]. Zou et al. [56] applying mannans from *Bacillus velenzensis* quantified an increased expression of antioxidant enzyme genes (Ascorbate peroxidase, Glutathione reductase and Glutathione-S transferase) in soybean plants with salt stress, showing that these mannans could act as an elicitor to improve plants resistance. This treatment could be a control measure not only in the vineyards, but also starting in nurseries to obtain better results in the vineyards [9].

## 5. Conclusions

In conclusion, asymptomatic vines of both cultivars responded to mannan treatment in a stress-like manner. Thus, in cv. Tempranillo, for both leaves and grapes almost all the parameters measured were affected, at least with some mannan treatment. The cv. Airén was less affected, especially in the parameters measured in leaves. For symptomatic vines, The measured biochemical processes and gene expression are less affected by the addition of mannans compared to asymptomatic vines. Mannans would act as a elicitor in asymptomatic vines since they caused stress triggering an increase in defense reactions that was not observed in symptomatic vines. The cultivar involved showed a different response to treatment with mannans. Likewise, in the cultivars studied, due to the degree of affectation of the grapes by the development of the disease, it was difficult to establish a correlation between the fruit and the leaf organ in the activity and results of gene expression of enzymes involved in the antioxidant system. According to these preliminary results, mannan treatment could be used, especially in asymptomatic vines, by activating their defense system to be able to respond more successfully to a possible infection of pathogenic fungi that trigger GTDs.

The use of mannans as a elicitor that can increase plant immunity would contribute to environmental conservation since they are extracted from renewable sources. In addition, these compounds are not expensive, making them a good alternative to reduce the use of pesticides and the impact of diseases on crops that cause large economic losses. However, it would be necessary to delve deeper into the real potential of these compounds, against diseases as complex as GTDs, in further studies.
